# Multislice CT angiography of the plantar arch

**DOI:** 10.2349/biij.6.1.e10

**Published:** 2010-01-01

**Authors:** L Field, Z Sun

**Affiliations:** Department of Imaging and Applied Physics, Curtin University of Technology, Perth, Australia

**Keywords:** plantar arch, computed tomography angiography, 3D visualization, hallux valgus, peripheral arteries

## Abstract

The aim of this case report is to present a multislice computed tomography angiography (CTA) procedure for viewing the plantar arch. A CTA was requested to determine the vascular sufficiency of the plantar arch of a 64-year-old patient with necrotic and gangrenous toes. The patient had recently undergone a proximal wedge osteotomy procedure for correction of a hallux valgus deformity. A 16-detector row CT scanner with 1.25 mm slice thickness and 0.625 mm reconstruction interval was used to reconstruct multiplanar reformats, maximum intensity projections and three-dimensional volume rendered images of the foot in question in both arterial and venous phases to determine if pathology of the plantar arch was present. The 3D reconstructed images of CTA demonstrated a loss of continuity of the plantar arch between the first and third metatarsals. This case report shows the diagnostic value of multislice CTA, especially 3D visualisation in the assessment of peripheral vascular branches.

## INTRODUCTION

Hallux valgus is a common joint deformity involving the first metatarsophalangeal joint resulting in a change in the cosmetic appearance of the foot and abnormal presentations such as pain and discomfort, depending on the degree of abnormality [1-3]. Blood supply to the lower extremity is extremely important, especially in patients with hallux valgus as the peripheral artery branches could be involved. The small plantar arch artery is very clinically significant in this abnormality because the necrotic toes are an indication of the death of cells and tissues as a consequence of reduced blood flow to that area. Traditionally, peripheral angiography is performed to investigate the main arteries of the lower limb and their branches; however, it is not only an invasive procedure but also associated with complications. With the advent of multislice computed tomography (MSCT), especially the 16- and 64-slice CT, large anatomic coverage can be easily achieved in a short time with high resolution images [4, 5].

Submillimetre CT scanning allows acquisition of nearly isotropic volume data with 16-slice CT (0.5×0.5×0.6 mm) and isotropic volume data with 64-slice CT (0.4×0.4×0.4 mm) [5, 6]. Thus, anatomic details such as peripheral blood vessels can be clearly demonstrated with MSCT angiography. In addition to the 2D axial images, high-resolution volume data permits generation of a series of 2D and 3D reconstruction images to enhance diagnostic value of MSCT for assessment of both normal vascular branches and abnormal changes [7, 8]. Of these reconstructions, multiplanar reformation (MPR), maximum-intensity projection (MIP) and volume rendering (VR) are most commonly used in clinical practice.

In this case report, the authors present an interesting patient with hallux valgus with necrotising toes. MSCT angiography images in 2D and 3D reconstructions were generated to demonstrate the diagnostic value of these images in the assessment of vascular supply to the peripheral arteries.

## PATIENT HISTORY

A 64-year-old man presented to the radiology department 8 days post hallux valgus corrective surgery. Due to the severity of the patient’s hallux valgus deformity, the surgery involved a proximal osteotomy of the first metatarsal with three k-wires, as well as pins inserted through the second, third and fourth metatarsophalangeal joints to maintain the bony alignment of these areas. After the surgery, the second and possibly third toe on the patient’s right foot had become necrotic and gangrenous. The medical team was querying the possibility of insufficiency of the plantar arch of the right foot, and requested an MRI to be performed on this foot. After examining the request form, a radiologist determined that a CTA procedure for the plantar arch would be more advisable in this situation due to the large amount of orthopaedic hardware present in the foot, but that an MRI would also be helpful to view the soft tissue areas of the foot after the CTA examination was performed.

## IMAGING GENERATION AND VISUALIZATION

The imaging examination was performed on a 16-detector row CT scanner (GE Lightspeed CT, GE Healthcare, Milwaukee, WI, USA) with the scanning protocol as follows: 120 kVp, 200-250 mAs, beam collimation 16 x 0.625 mm, gantry rotation time 500 ms, section thickness of 1.25 mm, pitch 1.375 and reconstruction interval of 0.625 mm. The area of scan coverage ranged from the lower third of tibia to the feet. During the procedure, 100 mL of a contrast medium (Isovue 370) was infused with 50 mL of saline and injected via a double power injector into the patient’s antecubital vein at a rate of 4 mL/s to visualise the vessels in and around the foot. A bolus tracking technique (Smart prep) of the thoracic abdominal aorta threshold of 100 HU over baseline was used to ensure maximum enhancement of the peripheral vessels. A scan delay of 60-70 seconds was used to demonstrate the venous supply to the lower extremity.

In addition to 2D axial views, a number of 3D reconstructions were generated to demonstrate the peripheral arteries. These reconstructions included multi-planar reformation (MPR), maximum-intensity projection (MIP) and three-dimensional volume rendered (3D VR) reconstructions of the foot. These particular reconstructions were used to effectively answer the clinical question and allow the plantar arch to be viewed as clearly as possible. Arterial and venous phase images were also produced to determine if the problem was due to arterial or venous insufficiency.

**Figure 1:**
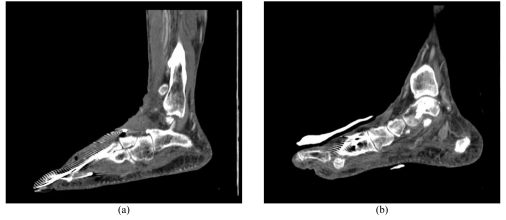
MPR view of the right plantar arch. Sagittal MPR images (A, B) demonstrate the plantar arch with apparent artifacts arising from the metal k- wires.

**Figure 2:**
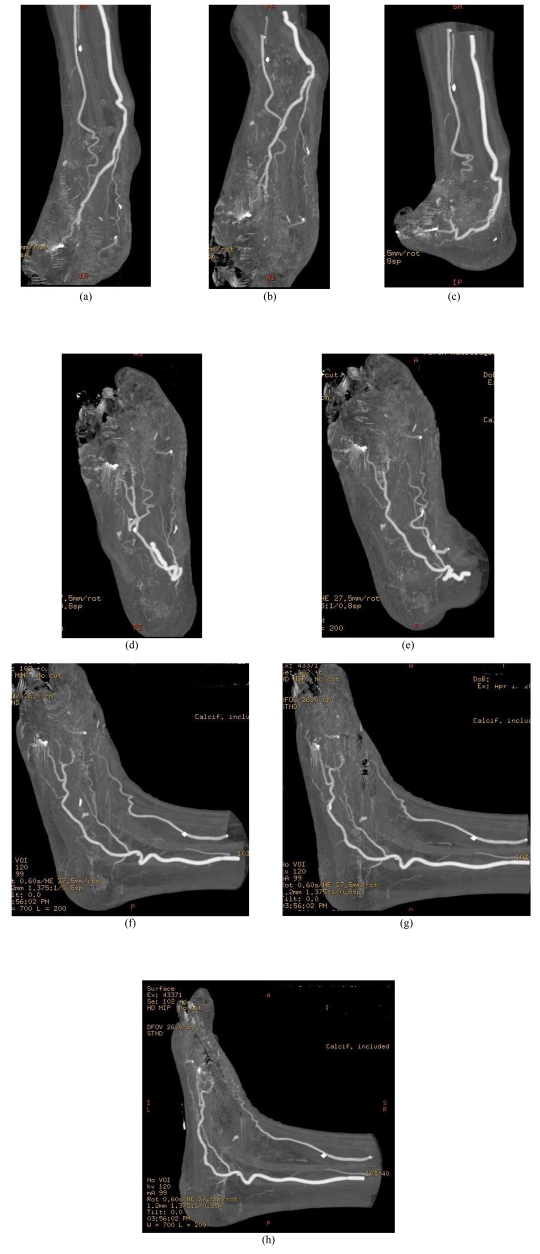
MIP visualisation of the plantar arch arteries. Coronal (A-D) and sagittal (E-H) MIP images show that the distal plantar arteries are occluded due to necrosis and occlusion in the location between 1^st^ and 3^rd^ metatarsal bones.

Figure 1 is a sagittal MPR view of the plantar arch, while Figure 2 is a series of MIP images showing the disruption of the plantar arch artery along its distal segment based on coronal and sagittal views.

**Figure 3:**
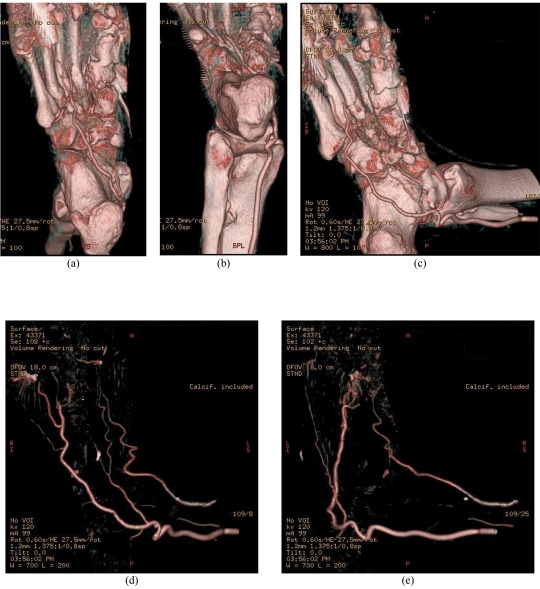
3D VR of the plantar arch arteries. VR images demonstrate the bony components and peripheral artery branches in the right foot (A-C), and the artery branches only (D, E). The distal plantar arteries between 1^st^ and 3^rd^ metatarsal bones are occluded.

3D VR images shade and colour pixels depending on their level of attenuation [9]. The VR reconstruction reveals surface and internal features, and allows the 3D images to be rotated in real time [10]. Figure 3 demonstrates 3D VR images of the vascular supply through the area of interest with and without bony components.

All of the above images demonstrate a loss of continuity of the plantar arch between the first and third metatarsal. The presence of the k-wires can also be seen by the metallic artifacts present on the images, particularly along the first metatarsal (Figs 2D, 3D).

## DISCUSSION AND CONCLUSION

Hallux valgus is a bony deformity that affects the first metatarsophalangeal joint. It involves a valgus movement of the proximal phalanx of the first metatarsal with an associated varus drift of the metatarsal head which can cause bursitis over the medial eminence of the first metatarsal with associated pain and discomfort [1]. Schweitzer et al [11] reported that the presence of an eminence, commonly referred to as a bunion, is seen in 95% of hallux valgus sufferers, while bursitis of the first metatarsal head is experienced in 70% of cases.

Radiographically, hallux valgus is assessed using weight- or load-bearing views of the affected foot, in order to determine the extent of loss of the transverse arch of the foot [12]. There is little to no use for multislice CT or MRI in diagnosing this disorder. Dorsi-plantar (DP), DP oblique and lateral weight-bearing radiographs are obtained, and measurements are taken off these radiographs to determine the severity of the disorder [3]. These measurements include the angle made between the first metatarsal and phalanges known as the hallux valgus angle, and the intermetatarsal angle, or the angle between the first and second metatarsals [11]. The main limitation of these measurements is the lack of providing vascular details in patients with suspected ischemic or necrotic changes.

MSCT allows acquisition of images much faster and with better quality and resolution than single-slice CT [4, 5, 13]. These technical improvements have significantly advanced the applications of MSCT, especially CT angiography (CTA) procedures. Schoepf et al [13] stated that MSCT allows CTA procedures to be used quickly and accurately for diagnosis of suspected vascular diseases in all organ systems. Clinical CTA applications are vast, and include examinations for the aorta, coronary arteries, carotid arteries, pulmonary vessels, vessels throughout the abdomen, brain and peripheral arteries [6-9, 14].

CTA of the peripheral arteries of the lower limb is an application that has been significantly enhanced with the development of MSCT technique as longer anatomic coverage is required to be obtained in a single breath-hold scan. Traditionally, examinations of this area required the use of invasive angiography to examine the run-off of the iliac and femoral arteries throughout the lower limb with the aim of assessing the presence of thrombus and atherosclerotic formations within the arteries branching directly from the iliac arteries [13]. However, the application of this technique in viewing the plantar arch is limited in the literature. Due to the invasiveness of conventional angiography, MSCT has become widely used in clinical practice as an effective alternative to invasive angiography for visualisation of vascular disease [15], including the peripheral artery branches, as shown in this case report.

While 2D axial CT images are routinely used in clinical practice, some kind of 2D or 3D reconstructions are required to provide information which is not available with 2D axial views, but still necessary for clinical requirements. MPR is the most commonly used complementary visualisation to 2D axial images as it allows quick demonstration of the relationship between anatomical structures. However, a number of MPR images are required to demonstrate the entire course of arteries and their branches, since not all of the vascular segments can be displayed in a single MPR view. This was observed in this case report (Fig 1), thus, application of MPR views in assessment of distal vascular territory, such as plantar arch is limited.

MIP has been widely recognized as the most useful visualisation tool in CT angiography as it provides angiographic-like images less invasively. The principle of MIP images is the demonstration of only maximum CT number encountered in each ray. The differentiation between contrast-enhanced blood vessels and background is good, thus high-density structures such as contrast-enhanced vessels and calcification can be clearly depicted and displayed on MIP images. MIP was found very useful in this case as it clearly shows the main arteries and their peripheral branches (Fig 2). However, MIP images do not provide depth information which is the main drawback for visualisation of complex anatomy.

3D VR provides a 3D representation of the anatomical structures based on a volume dataset, since it utilises all of the information contained in the data. A voxel-based intensity histogram is generated, and several parameters such as colour, brightness and opacity are assigned to each voxel according to its CT attenuation value. Therefore, 3D relationship between different structures can be easily displayed and appreciated on VR, as shown in Figure 3. VR allows demonstration of both vascular branches and bony components in a single image, thus it significantly enhances understanding of the complex anatomic structures. VR requires extensive user interaction for accurate evaluation of complex anatomical structures. Currently, advanced 3D post-processing software packages are available in most of the recent MSCT scanners, so the VR display of these complex structures including peripheral arteries becomes feasible without further segmentation. VR image quality is determined by the resolution of original source data.

In this case study, radiologists were specifically looking for evidence to confirm the lack of presence of the plantar arch, a relatively small artery located deep within the foot. While MIP images provide necessary information about the patency of the plantar arch arteries, the use of 3D VR reconstructions through the plantar arch were extremely important to diagnose and confirm that the continuity of the plantar arch had indeed been lost and blood flow to the digits of the foot had been compromised. These reconstructions allowed both internal and surface features to be seen and allowed all images to be rotated in real time. Without using 3D VR reconstructions, determining the sufficiency of the plantar arch in this case would have been almost impossible and the patient’s long-term health and quality of life would have been significantly affected.

In conclusion, the authors have demonstrated the application and usefulness of MSCT angiography in the assessment of vascular supplies to the small plantar artery in a patient diagnosed with hallux valgus. A combination of 2D and 3D reconstructions of MSCT angiography (a combination of MIP and VR) proved to be valuable in confirming the perfusion of peripheral artery branches.

## References

[1] Lin JS, Bustillo J (2007). Surgical treatment of hallux valgus: a review. Curr Opin Orthop.

[2] Hart ES, deAsla RJ, Grottkau BE (2008). Current concepts in the treatment of hallux valgus. Orthop Nurs.

[3] Sammarco GJ, Idusuyi OB (2001). Complications after surgery of the hallux. Clin Orthop Relat Res.

[4] McCollough CH, Zink FE (1999). Performance evaluation of a multi-slice CT system. Med Phys.

[5] Sun Z, Lin C, Davidson R (2008). Diagnostic value of 64-slice CT angiography in coronary artery disease: a systematic review. Eur J Radiol.

[6] Sun Z, Jiang W (2006). Diagnostic value of multislice computed tomography angiography in coronary artery disease: a meta-analysis. Eur J Radiol.

[7] Sun Z (2009). Helical CT angiography of fenestrated stent grafting of abdominal aortic aneurysms. Biomed Imaging Interv J.

[8] Sun Z, Mwipatayi BP, Allen YB (2009). Multislice CT angiography of fenestrated endovascular stent grafting for treating abdominal aortic aneurysms: a pictorial review of the 2D/3D visualizations. Korean J Radiol.

[9] Kalra MK, Saini S, Rubin GD (2008). MDCT: From Protocols to Practice.

[10] Duddalwar VA (2004). Multislice CT angiography: a practical guide to CT angiography in vascular imaging and intervention. Br J Radiol.

[11] Schweitzer ME, Maheshwari S, Shabshin N (1999). Hallux valgus and hallux rigidus: MRI findings. Clin Imaging.

[12] Suzuki J, Tanaka Y, Takaoka T (2004). Axial radiographic evaluation in hallux valgus: evaluation of the transverse arch in the forefoot. J Orthop Sci.

[13] Schoepf UJ, Becker CR, Hofmann LK (2003). Multislice CT angiography. Eur Radiol.

[14] Hofer M (2005). CT Teaching Manual: a systematic approach to CT reading.

[15] Sun Z (2006). Diagnostic accuracy of multislice CT angiography in peripheral arterial disease. J Vasc Interv Radiol.

